# Piloting a new cross-sector model of care to support parents with cancer: feasibility and acceptability of the Parent Support Worker role

**DOI:** 10.1007/s00520-024-08629-6

**Published:** 2024-06-15

**Authors:** X. Skrabal Ross, S. Konings, E. Schiena, J. Phipps-Nelson, Y. Wang, F. Hodgson, P. Patterson, F. E. J. McDonald

**Affiliations:** 1 Policy and Patient Department, Canteen Australia, Sydney, Australia; 2 Cancer and Palliative Care Outcomes Centre, Queensland University of Technology, Brisbane, Australia; 3 Psycho-Oncology Department, Clinique Saint-Jean, Brussels, Belgium; 4 Department of Allied Health, Peter MacCallum Cancer Centre, Melbourne, Australia; 5 Department of Oncology, Sir Peter MacCallum, University of Melbourne, Melbourne, Australia; 6 Office of Cancer Research, Peter MacCallum Cancer Centre, Melbourne, Australia; 7 Guangxi Minzu University, Nanning, China; 8 John Hunter Hospital, Newcastle, Australia; 9 Faculty of Medicine and Health, The University of Sydney, Sydney, Australia

**Keywords:** Oncology, Parental cancer, Adolescents, Young adults, Family support, Health systems

## Abstract

**Purpose:**

A new Parent Support Worker (PSW) service was piloted in three Australian hospitals. This study assesses the feasibility and acceptability (including preliminary effectiveness) of the service in supporting cancer patients with children.

**Methods:**

A multi-site, mixed-methods study collected quantitative and qualitative data on the effectiveness of the service (pre post-test, *n* = 36), qualitative and quantitative data on acceptability of the service (survey, *n* = 43), and qualitative data on acceptability (semi-structured interviews, *n* = 13). Feasibility was assessed through rates of service uptake amongst referred parents.

**Results:**

Of 1133 parents referred, 810 (71%) accepted to receive the service, suggesting high interest in PSW support. Interviewees likewise reported that the service was accessible and facilitated further referrals, indicating good feasibility. Surveys completed three months after accessing PSW support showed high acceptability and satisfaction. Additionally, there was preliminary evidence of service impacts: parents’ distress, parenting concerns, parenting efficacy, and stress about situations of concern improved significantly from pre- to post-service (all *p* < 0.005). Interviewees further described how their emotional coping and confidence to support and communicate with their children had improved through contact with the service.

**Conclusion:**

The PSW service, integrated into a novel cross-sector model of care, showed to be feasible and acceptable to parent patients and their partners and improved psychological and parenting outcomes. The study suggests refinements to the service and the need for future larger studies to explore the effectiveness of the service in improving parents’ outcomes. This study complements previous evidence on the implementation of the PSW service in hospitals.

**Supplementary Information:**

The online version contains supplementary material available at 10.1007/s00520-024-08629-6.

## Introduction

Parental cancer significantly impacts family functioning, imposing challenges on parents and children [1, 2]. Diagnosed parents grapple with the responsibilities of managing their illness and treatment while fulfilling parental responsibilities [3, 4]. Prioritizing their children’s well-being often leads to greater anxiety and depression compared to patients without dependent children [5, 6]. Communicating the diagnosis and treatment with their children becomes a major stressor. Parents worry about potential effects on their children’s psychosocial well-being [7, 8]. Observing behavioral or emotional changes in their children may induce feelings of parental guilt [5].

A parental cancer experience can negatively affect children’s mental, emotional, and physical well-being [1, 9]. Adolescents and young adults (AYAs, 12–25 years old) are particularly vulnerable due to the significant cognitive, social, and emotional development that occurs during this critical life stage. Consequently, AYAs tend to experience higher distress, anxiety, worry, and concentration difficulties compared to younger children with a parent diagnosed with cancer [10, 11].

Addressing the psychosocial needs of parents with cancer and their families is crucial for coping, adapting, and maintaining overall well-being [12, 13]. Unfortunately, standard oncology services often overlook these specific psychosocial needs [14]. Health professionals may lack awareness of the importance of psychological support in families with dependent children, often focusing solely on the physical aspects of cancer [15]. Limited experience, emotional challenges, time constraints, and uncertainty surrounding professional roles may hinder health professionals from providing emotional support to parent patients and their AYA children [12, 14, 16, 17].

While the need for educational and psychological interventions to support families coping with parental cancer is recognized [18, 19], evidence on interventions, is limited [4, 11, 20]. Several interventions have demonstrated effectiveness in enhancing family outcomes: For example, improved parental knowledge about age-appropriate cancer information, increased confidence around communicating and parenting, improved parenting skills, family resilience, parental depression, and children’s behavioral and emotional adjustment [8, 18, 19, 21]. However, these interventions are rarely embedded in standard practices, leaving families with limited access [2, 9].

The Parent Support Worker (PSW) service, as part of a novel cross-sector model of care, was piloted in three Australian hospitals to provide integrated psychosocial care to parents with cancer, their partners, and children. Jointly funded by philanthropic funding and hospital support, the initiative placed dedicated social workers in adult oncology wards. These PSWs, worked alongside hospital social work teams to offer practical, psychological, and emotional support to parent patients and their families; implementation and role characteristics varied in accordance with hospital contexts and funding requirements (described in Skrabal Ross et al. [14]). Evaluation work was subsequently undertaken to assess the implementation, feasibility, and acceptability of the PSW role, with the potential to inform decisions about the continuation, expansion or revision of the service. Proctor and colleagues’ implementation framework [22] was used to guide the operationalization of these constructs, which were assessed from both service providers’ and service users’ perspectives.

Findings from service providers’ perspectives indicated high feasibility, acceptability, and appropriateness of the PSW role: PSWs were seen to reduce existing staff workload while enhancing specialized psychosocial services to cancer-affected families with children [14].

This research aims to complement healthcare professionals’ perspectives on the PSW role by exploring the feasibility and acceptability of the PSW service amongst parent patients with young and AYA children and their partners. Additionally, the study aims to explore the preliminary effectiveness of the PSW service in improving psychological and parenting outcomes for parent patients.

## Methods

### Study design

This multi-site study employed a mixed-methods design, guided by Proctor and colleagues’ implementation framework [22]: (1) service uptake was calculated to assess feasibility; (2) post-service and satisfaction surveys explored service acceptability; (3) pre/post-surveys assessed changes in parenting and psychological outcomes after 3-month engagement with the PSW service; and (4) 30-min semi-structured phone interviews with a subset of parents asked about the acceptability and impact of the service. Convenience sampling was employed to recruit participants; due to differences in PSW role implementation between hospitals, not all methods were employed at all sites. Access to the service was not conditional on completing the study.

This study was approved by the Peter MacCallum Cancer Centre Human Research Ethics Committee (PMHREC) (HREC/17/PMCC/210). Governance approvals were obtained from hospital sites: Hunter New England Research Ethics & Governance Office (SSA/18/HNE/122), South Western Sydney Local Health District (SSA/18/LPOOL/259), and PMHREC (SSA/18/PMCC/12).

### Participants and procedure

Participation was open to adult cancer patients (and/or their partners) with young or AYA children, who accessed the PSW service through the three Australian hospitals.

#### Feasibility

Feasibility data was collected by H1 (the largest site), operationalized as participation rates amongst parents referred to the PSW service. Reasons for non-participation were also asked. This was not possible in the other hospitals due to internal processes.

#### Pre/post-service surveys

On booking their first PSW sessions, hospital 1 (H1) participants received a study information brochure and the pre-service survey, including demographic and clinical characteristics and outcome measures (distress, parenting concerns, parental efficacy). Three months after the first session, participants completed the post-service survey, comprising outcome measures and brief questions about service acceptability.

#### Satisfaction surveys

Satisfaction surveys were provided to H1 clients who could not complete the pre-service survey, and (alongside study information brochure) to all clients of hospitals 2 and 3 (H2, H3) who for logistical reasons could not complete pre-service surveys. These surveys collected demographic and clinical information, as well as service acceptability data.

All surveys could be completed online or on paper (sealed envelope provided for confidentiality). Online surveys were emailed to participants automatically and printed surveys were provided and collected through non-PSW hospital staff. Consent was considered implicit in survey completion/return.

#### Interviews

At the end of the post-service and satisfaction surveys, participants could anonymously indicate interest in participating in an interview. Interviews elicited further detail about service acceptability and impact on participants and their families. Consent was assumed from participation.

### Survey measures

#### Distress

The Kessler-10 (K10) assessed psychological distress [23]. It comprises 10 items on a 5-point scale ranging from (1 = never–5 = all the time). Total scores range from 10 to 50, classified as low (10–15), moderate (16–21), high (22–29), or very high distress (30–50). The K10 has excellent internal consistency (Cronbach’s *α* = 0.93) and is widely used in international and Australian health contexts [24].

#### Parenting concerns

The 15-item Parenting Concerns Questionnaire–General (henceforth, PCQ-General) measured parenting-related distress on three subscales: practical impact, emotional impact, and co-parent [25]. Participants respond using a 5-point scale (not at all concerned–extremely concerned) with higher total scores indicating more parenting concerns. The PCQ demonstrates good internal consistency (Cronbach’s *α* = 0.83–0.93), and convergent validity with measures of distress [25, 26].

This was supplemented by the Parents’ Concerns Questionnaire V9-Specific (initial form), a clinical tool used for assessing presenting concerns of parents seeking support^1^ (henceforth, PCQ-Presenting Concerns). Two open-ended questions ask about situations/behaviors participants would like help with; each situation/behavior is followed by three questions about frequency (1 = once or twice a month–7 = 5 or more times a day) and parents’ and children’s stress about the situation/behavior (1 = not stressed at all–7 = extremely stressed). There is no published information on its validity as it was designed and used as a clinical scale.

#### Parental efficacy

The Help-Child subscale of the Cancer Self-Efficacy Scale (CASE; 9 items)[18] assesses parental efficacy to support children through cancer. Parents rate statements on a 10-point scale (1 = not at all confident–10 = very confident), higher scores indicating greater parental efficacy. It has good internal consistency (Cronbach’s alpha = 0.93). Higher Help-Child scores are associated with lower maternal anxiety and depression and fewer child internalizing/externalizing behaviors.

##### Service acceptability

Post-service and satisfaction surveys included nine questions about service acceptability (helpfulness, satisfaction, most beneficial aspects, areas for improvement).

### Data analysis

Quantitative survey data were analyzed using SPSS V27 (IBM SPSS Inc., Chicago, IL, USA). Sample demographic/clinical characteristics and quantitative feasibility and acceptability data were presented using descriptive statistics. Chi-squared or Fisher’s exact tests or Pearson’s correlation coefficient were used to compare socio-demographic and clinical characteristics of participants who only completed pre-service surveys and those who completed both pre and post-surveys. Pre-post differences in levels of distress and parenting confidence and concerns were compared using paired samples *t* tests or Wilcoxon signed rank tests.

Qualitative survey and interview data were analyzed using qualitative content analysis [27, 28]. An essentialist/realist methodology was employed, using an inductive approach [29]. This meant themes were closely linked with the data, situating thematic meaning at the semantic or surface level of the data. Themes were defined as common ideas present within the data that were relevant to the objectives of the study, which influenced the viewpoint for examination of the data, with a variable oriented-approach aiming to find common elements instead of individual experiences [30]. Iterative coding was employed by returning to the data several times to check for relevance and commonality of the codes. Data were coded by XSR and reviewed by FEJM for agreement.

## Results

### Pre/post-service survey participants

Participants completing pre/post-service surveys (*n* = 36) and those completing only pre-service surveys (*n* = 30) did not differ significantly in demographics, including gender (Fisher’s exact test, *p* = 0.693), age (*r* = 23) = (29.25, *p* = 0.172), languages spoken at home (*r* = 1) = (0.14, *p* = 0.700), birth country (*r* = 1) = (0.24, *p* = 0.877), and if they lived with their partner (*r* = 1) = (0.98, *p* = 0.754). Parent-patients who only completed the pre-service survey were more likely to report accessing mental health services for cancer-related support before accessing the PSW service (*r* = 1) = (8.73, *p* = 0.003).

Parents (all patient-parents) completing pre- and post- service surveys were mostly female (92%), 36–55 years old (66%), living with their partner (86%), and had not previously accessed cancer-related mental health services (71%). More than half had two children (56%), and most (89%) had attended one or two PSW sessions at the time of the post-service survey (Table 1).

**Table 1:** Demographic and cancer characteristics of the pre-post service survey participants

Demographic and cancer characteristics	*n*	%
**Gender**		
Male	3	8%
Female	33	92%
**Age (years)**		
26–35	8	22%
36–45	16	44%
46–55	11	31%
56–65	1	3%%
**Country of birth**		
Australia	27	75%
Other	9	25%
**Speak a language different from English at home**		
Yes	7	19%
No	29	81%
**Employment**		
Full-time, part-time, casual	28	78%
Not employed or unpaid leave	8	22%
**Number of children**		
1	6	17%
2	20	56%
3	8	22%
4	2	5%
**Live with partner**		
Yes	31	86%
No	5	14%
Accessed cancer-related mental health support prior to contact with PSW *		
Yes	10	29%
No	25	71%
**Diagnosed with cancer (cancer patient)**		
Yes	36	100%
**Number of sessions with the PSW (at post-service survey)**		
1	22	61%
2	10	28%
3	2	6%
4	2	6%

*Missing answer = 1

### Satisfaction survey participants

Forty-three parents completed satisfaction surveys; 61% were female, 49% were aged 46–55 years and 54% had 2 children (range 1–5), 59% were cancer patients, and 8% spoke languages other than English. On average, they had completed 2.6 (range 1–10) PSW sessions by the time of participation.

### Feasibility of the PSW service

Between May 2018 and June 2022, 1133 parents at H1 were referred to the PSW service by the hospital and 810 (71%) accepted to receive the service. The most common reason for non-participation was not needing the service, followed by death.

### Effectiveness of the PSW service

#### Psychological distress

Parents’ distress decreased significantly from pre-service (*M* = 22.7, high distress) to post-service (*M* = 19.4, moderate distress) (*t*(35) = 3.453, *p* = 0.001).

**Fig. 1:**
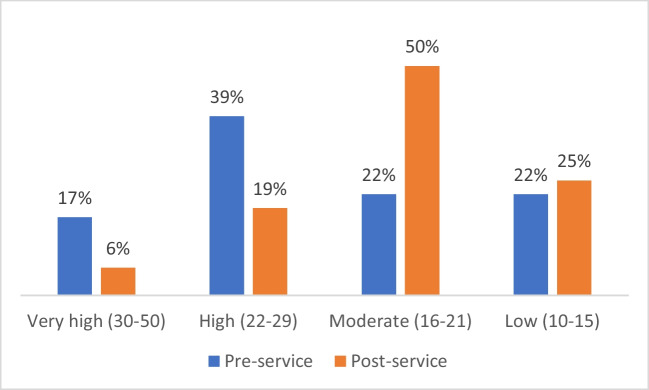
Sample’s levels of distress at pre and post-service

The proportion of parents with high distress was halved at post-service (39% vs. 19%), and those with very high distress was almost 3 times lower (17% vs. 6%). Figure 1 shows distress levels at pre- and post-service assessment.

#### Parenting concerns and efficacy

##### PCQ-general

Total parenting concerns decreased significantly from pre- (*M* = 2.9) to post-service (*M* = 2.3;* t*(35) = 4.949, *p* < 0.001). However, both means reflect similar levels of concern (somewhat concerned) according to Inhersten’s classification [26]. The practical impact subscale showed the highest levels of concern at pre-service (*M* = 3.6, “very concerned”) and was significantly reduced at post-service (*M* = 2.8, “somewhat concerned”; *t*(34) = 4.320, *p* < 0.001). The emotional impact and co-parenting concerns subscales were also significantly reduced at post-service (emotional impact, mean 2.8 vs. 2.3; *t*(34), *p* < 0.001; co-parenting, mean 2.1 vs. 1.8, *t*(32), *p* = 0.026). Changes in specific items of the PCQ-General are presented in Supplementary Material 1.

##### PCQ-presenting concerns

Parents’ stress about the situations they hoped to improve with the service significantly decreased from pre- to post-service, situation 1 (*M* 5.6 vs. 3.9; *t*(25) = 4.623, *p* = 0.001), situation 2 (*M* 5.3 vs. 3.9; *t*(23) = 3.300, *p* = 0.003). However, parent-perceived children’s stress about these situations did not significantly decrease.

At post-service, 73% of parents agreed that the situations they wanted to improve with the PSW services “got better”.

##### Parental efficacy

Parents’ perceived self-efficacy to support children with cancer-related concerns increased significantly from pre- (*M* = 47.7) to post-service (*M* = 62.8; *t*(33) = 4.325,* p* = 0.001).

### Acceptability of the PSW service

Participants completing satisfaction or post-service surveys were mostly or very satisfied with the PSW service: the majority said that the service helped (88%) including with talking to children about cancer (86%) and feeling more confident parenting through cancer (92%). Most said the service was easy to access and they would recommend it to other parents with cancer (97%) and 65% received help with more than one support need, with the main being help with managing feelings (61%), practical issues (49%), and managing parenting (44%).

#### Interviews

Thirteen participants completed interviews (no demographic data available). Five themes were identified, described below.

1. Improved confidence to communicate with and support children.

Parents expressed that PSWs provided them with information, resources, and consultation that increased their confidence in communicating with their children about cancer, and gave them strategies to support their children to manage the impact of parental cancer.

“I didn’t want to tell them [children] too soon and I didn’t want to scare them, but then I also didn’t want to withhold information from them. So that’s where [PSW] came into helping me decide what was the appropriate amount of information for all of them” P4

2. Improved emotional coping.

PSWs helped parents to manage emotions related to the cancer diagnosis and treatment, including by normalizing reactions and helping them to feel calmer about their experience.

“[PSW] just helped me to see a bit more realistic view of what was going to happen and how things were going to progress. And so I was able to be a little bit calmer about it all, not quite so anxious about it all” P4

3. Facilitated referrals.

PSWs referred parents to services appropriate for their own, partners’ and children’s support needs (e.g., psychologist, musical therapy) and liaised with service providers to facilitate access.

“It gave me some resources, websites, and she also referred me to the psychologist at [hospital] for my own needs… My husband and I too were having some issues. She also suggested some other programs at [hospital]. For example, there was music therapy they were starting up for children. She gave me resources for Canteen” P1

4. Easily accessible.

Parents expressed that appointments were easily booked and convenient to access (including hospital-based, home visits or remote sessions).

“It was quite easy to make an appointment - well, we were referred [PSW] got in contact with us and then we booked it up, and it was on the same day as treatment” P11

5. Needed further follow-ups.

New cancer-related support needs could arise after initial needs were met. Parents expressed their desire for more follow-ups from PSWs and their preference for the service to contact them to identify and discuss new support needs.

“The way that [PSW] contacted me was a great thing, but I think it should have continued. Were there other organisations that I can access? I’m assuming that it would be the same as what [PSW] did, but I don’t think it should come down to the cancer patient to be – to contact them. I think there needs to be something that opens the door for the cancer patient” P13

## Discussion

In evaluating a novel, integrated, and holistic cross-sector model of care, this study contributes to the limited evidence on health services supporting families impacted by parental cancer. This pilot evaluation of the PSW service suggested that the role is feasible and acceptable to parents using the service; moreover, it provided preliminary evidence that service use is associated with decreased distress, decreased parenting concerns, and improved parental efficacy. Together with feedback from PSWs and healthcare professionals [14], this is a promising indication of the potential for the PSW role to provide integrated psychosocial support to families impacted by parental cancer—particularly in a landscape where many parents (71% in this study) have not previously accessed cancer-related mental health support.

Service providers’ perspectives on the PSW service suggested that the service was feasible, with service activity data indicating that the three PSWs were able to support 630 families through 1243 sessions and 142 secondary consultations across a 2-year period [14]. Data from this paper complements previous findings, showing high service uptake amongst referred parents. Moreover, the top reason for non-use was lack of need, suggesting that uptake may be higher amongst parents in distress or with unmet needs; however, there may be a need for integration or referral to bereavement services, as death was the second most common reason for non-use. Survey and interview data from parents indicated that the service was easily accessible, convenient, and flexible—although more follow-up is desired to identify and address subsequent needs.

Building on previous work demonstrating that the PSW service is acceptable and appropriate to hospital staff [14], this study confirms that the service is also acceptable to parent-patients and partners. Most participants reported being satisfied and agreed that the service had helped them. Specifically, parents indicated that PSWs helped improve parenting-related issues that are not routinely identified and addressed by oncology healthcare systems, for example communication with their children about cancer and management of practical and emotional aspects of cancer related to their parenting role.

Findings from the post-service and satisfaction surveys are echoed by results from the pre/post-service part of the evaluation, which showed statistically significant decreases in distress and parenting concerns (on both measures), coupled with an increase in parental efficacy. This is particularly promising as previous studies have shown parents with cancer to experience high rates of mental health concerns [31–35], with 60% in this study reporting high/very high levels of distress. Interviews suggested that this may be partly due to improved emotional coping. While the PSW service halved the number of parents with high and very high distress levels, 25% of parents remained highly distressed after 3 months. Likewise, while the PSW service addressed areas of high concern, at post-service parents remained highly concerned about the impact of their mood and physical limitations on their children. The provision of tailored and ongoing support to parents with high distress, or persistent concerns about how their mood or physical limitations affect children, should be considered in future improvements to the service.

Interviewees described feeling more confident in communicating with and supporting their children. Likewise, the PSW service significantly improved parental confidence in supporting their children with cancer-related concerns at post-service (47.7 vs. 62.8); however, there is room for further improvement (maximum levels = 90). As baseline levels of parental confidence in this study were lower than those found by previous studies [18, 36], it is possible that more PSW sessions were needed for parents to improve their confidence in helping their children. Similarly, parents in this study expressed the need for more follow-ups from PSWs after their initial issues improve, as cancer is a dynamic experience and new issues may arise that require support; future refinements to the service may be required to extend the provision of the support based on parents outcomes and needs. This may include the adoption of systematic assessment of parents’ distress and needs at multiple time points and the use of qualitative methods to gather information about parents’ additional concerns and stressors that may affect their lives and family relations. Future evaluations of the service should include an extended follow-up period accordingly.

It is worth noting that parents’ perceptions of their children’s stress levels did not improve significantly between pre- and post-service surveys. It is not uncommon for a service to not demonstrate improvements over all investigated outcomes, and given the ongoing parental cancer situation, it is perhaps not unexpected for children’s stress levels to remain high. Future evaluations may benefit from incorporating measures that may be more sensitive to change, such as unmet needs [37], or incorporating child-reported outcomes, as discrepancies between parent- and child-reported wellbeing have been previously reported [9].

### Implications

Despite of the high feasibility and acceptability of the service, this study highlights opportunities for refinement. Co-creation with service end-users may increase the relevance of the service; it is recommended that parent patients are involved in ideas and decisions about future changes to the service and its evaluation from early stages, which was not possible in the present study.

This work has important implications for health service delivery. Preliminary evidence on the effectiveness of the PSW service shows that the service has the potential to improve parent patients’ psychological and parenting outcomes, which may translate into improved wellbeing for their children. Furthermore, this model of care is likely to support and reduce the burden on healthcare systems in meeting psychological and parenting needs of parent patients in a streamlined and coordinated way [14]. The PSW role may also be effective in parent populations affected by other life-threatening chronic diseases which present similar challenges to cancer (e.g., severe side-effects, long hospitalizations) with high practical and emotional impact on their children and family.

### Limitations

Care should be taken when generalizing findings from this study, considering the low proportion of service users participating (with only 4.4% of service users at H1 completing pre- and post-service measures) and limited representation of men and non-patient parents. Participants’ self-selection to provide feedback may mean that reports of positive experiences are overrepresented (response bias; e.g., [38]), or that findings may not be true for underrepresented groups (e.g., men). As a non-controlled pilot study, this evaluation was not able to delineate service impacts from expected fluctuations or improvements in wellbeing over time; likewise, its scope was limited to investigating overall changes in psychosocial and parenting outcomes, rather than exploring how this may be moderated by demographic or cancer characteristics. For example, families impacted by advanced or terminal cancer might have higher distress, unique parenting concerns, or different outcome trajectories over time, potentially necessitating tailored services; the experiences and impacts for this population warrants further exploration.

The design and methodology employed by this study were unavoidably limited by the funding and hospital systems in which the service operated, which necessitated the rapid design and implementation of the service and its evaluation and constrained the ability to implement more rigorous methods. For example, only one hospital had an appointment booking system which allowed pre-service surveys to be sent and completed before meeting the PSW, and only one hospital was able to collect feasibility data. Likewise, as a non-controlled trial (as it was considered unethical to deny or delay support services to parents in need), this study was unable to delineate service impacts from expected natural adaptation over time. The PSW service and its evaluation should thus be considered in the context of this constrained system, as an example of the compromises between rigor and practicality that are often necessary in health service research. However, the inclusion of multiple methods and stakeholder perspectives in the evaluation is intended to compensate for this to some extent, providing a more comprehensive picture of service implementation, experiences, and outcomes.

## Conclusions

Findings from this study support the feasibility and acceptability of the PSW service to patients and partners as well as its potential to improve parent patients’ psychological distress, parents’ concerns, efficacy, and stress about the situations they sought help for. The study highlights areas where the service can be improved to enhance support to parents with cancer. It complements previous evidence on the implementation of the PSW service in hospitals, which showed high acceptability and appropriateness to hospital staff and feasibility for the service to be implemented. The model of care supports healthcare systems in addressing parent patients psychological and parenting needs in a streamlined and coordinated way. Future larger research is required to explore the effectiveness of the service in improving psychological and parenting outcomes in parents with cancer and their partners.

Authors’ contribution statements.

X Skrabal Ross (data collection, designed and conducted data analysis, manuscript draft, manuscript revision, and final approval), S Konings (data collection, manuscript revision and final approval), E Schiena (data collection, manuscript revision, and final approval), J Phipps-Nelson (data collection, manuscript revision, and final approval), Y Wang (manuscript draft, revision, and final approval) F Hodgson (data collection, manuscript revision, and final approval), P Patterson (study design, manuscript revision, and final approval), FEJ McDonald (senior author, study design, data collection, manuscript revision, and final approval).

### Supplementary Information

Below is the link to the electronic supplementary material.

Supplementary file1 (DOCX 23 KB)

## Data Availability

The datasets generated and analyzed during the current study are not publicly available due to containing information that could compromise the privacy of research participants but are available from the corresponding author on reasonable request.

## Footnotes

1 Developed by S. Matthey and A. White from South West Sydney Area Health Service, Sydney, Australia.
