# Supplementary material for: Piloting a new cross-sector model of care to support parents with cancer: feasibility and acceptability of the Parent Support Worker role
Source: Support Care Cancer. 2024 Jun 15;32(7):435. doi: 10.1007/s00520-024-08629-6 (PMC11180003; doi:10.1007/s00520-024-08629-6)
Supplement: Supplementary file 1 — Supplementary file1 (DOCX 23 KB) [file 520_2024_8629_MOESM1_ESM.docx]

**Supplementary File 1. Sample’s average scores on individual items on the Parenting Concerns Questionnaire – General (PCQ) measure at pre-service and post-service**

| **Item** | **Pre-service** | **Post-service** |
| --- | --- | --- |
|  | Average (SD) | Average (SD) |
| **Practical Impact** |  |  |
| My own mood, worries or emotions are affecting my children | 4.03 (0.97) | 3.33 (1.28) |
| My physical limits or low energy are affecting my children | 4.00 (0.86) | 3.17 (1.32) |
| I am not able to spend as much time with children as I would like | 3.66 (1.18) | 2.80 (1.32) |
| The illness is changing my children’s routine | 3.37 (1.06) | 2.47 (1.40) |
| Changes in my memory or attention are affecting my children | 3.14 (1.24) | 2.11 (1.14) |
| **Emotional Impact** |  |  |
| My children are emotionally upset by the illness | 3.53 (1.23) | 2.78 (1.31) |
| My children are worried that I am (or my partner is) going to die | 3.09 (1.38) | 2.36 (1.39) |
| My children get upset when we talk about the illness | 2.80 (1.30) | 2.19 (1.09) |
| My children might be in need of professional healthcare | 2.50 (1.37) | 2.03 (1.25) |
| My children get confused or upset by what others say about the illness | 2.31 (1.05) | 1.97 (1.23) |
| **Concerns about the Co-Parent** |  |  |
| My children’s other parent would not be able to meet their emotional needs if I’m unable to | 2.85 (1.37) | 2.49 (1.26) |
| There is no one to take good care of my children if I am unable to | 2.03 (1.14) | 1.67 (0.98) |
| My partner is not providing me with enough practical support | 1.91 (1.15) | 1.62 (0.92) |
| My partner is not providing me with enough emotional support | 2.21 (1.24) | 1.91 (1.13) |
| My children’s other parent would not be a responsible caregiver if I were unable | 1.71 (1.08) | 1.71 (1.07) |
